# Epidemiology of bacterial co-infections and risk factors in COVID-19-hospitalized patients in Spain: a nationwide study

**DOI:** 10.1093/eurpub/ckad060

**Published:** 2023-04-22

**Authors:** R López-Herrero, L Sánchez-de Prada, A Tamayo-Velasco, M Lorenzo-López, E Gómez-Pesquera, B Sánchez-Quirós, O de la Varga-Martínez, E Gómez-Sánchez, S Resino, E Tamayo, A Álvaro-Meca

**Affiliations:** BioCritic, Group for Biomedical Research in Critical Care Medicine, Valladolid, Spain; Anesthesiology and Critical Care Department, Hospital Clínico Universitario de Valladolid, Valladolid, Spain; Department of Surgery, Faculty of Medicine, Universidad de Valladolid, Valladolid, Spain; BioCritic, Group for Biomedical Research in Critical Care Medicine, Valladolid, Spain; Microbiology and Immunology Department, Hospital Clínico Universitario de Valladolid, Valladolid, Spain; BioCritic, Group for Biomedical Research in Critical Care Medicine, Valladolid, Spain; Haematology and Hemotherapy Department, Hospital Clínico Universitario de Valladolid, Valladolid, Spain; BioCritic, Group for Biomedical Research in Critical Care Medicine, Valladolid, Spain; Anesthesiology and Critical Care Department, Hospital Clínico Universitario de Valladolid, Valladolid, Spain; Department of Surgery, Faculty of Medicine, Universidad de Valladolid, Valladolid, Spain; BioCritic, Group for Biomedical Research in Critical Care Medicine, Valladolid, Spain; Anesthesiology and Critical Care Department, Hospital Clínico Universitario de Valladolid, Valladolid, Spain; Department of Surgery, Faculty of Medicine, Universidad de Valladolid, Valladolid, Spain; BioCritic, Group for Biomedical Research in Critical Care Medicine, Valladolid, Spain; Anesthesiology and Critical Care Department, Hospital Clínico Universitario de Valladolid, Valladolid, Spain; BioCritic, Group for Biomedical Research in Critical Care Medicine, Valladolid, Spain; Department of Anesthesiology, Hospital Universitario Infanta Leonor, Madrid, Spain; BioCritic, Group for Biomedical Research in Critical Care Medicine, Valladolid, Spain; Anesthesiology and Critical Care Department, Hospital Clínico Universitario de Valladolid, Valladolid, Spain; Department of Surgery, Faculty of Medicine, Universidad de Valladolid, Valladolid, Spain; Unidad de Infección Viral e Inmunidad, Centro Nacional de Microbiología, Instituto de Salud Carlos III, Madrid, Spain; BioCritic, Group for Biomedical Research in Critical Care Medicine, Valladolid, Spain; Anesthesiology and Critical Care Department, Hospital Clínico Universitario de Valladolid, Valladolid, Spain; Department of Surgery, Faculty of Medicine, Universidad de Valladolid, Valladolid, Spain; Departament of Preventive Medicine and Public Health, Faculty of Health Science, Universidad Rey Juan Carlos, Madrid, Spain; Centro de Investigación Biomédica en Red de Enfermedades Infecciosas (CIBERINFEC), Instituto de Salud Carlos III, Madrid, Spain

## Abstract

**Background:**

We performed a nationwide population-based retrospective study to describe the epidemiology of bacterial co-infections in coronavirus disease 2019 (COVID-19)-hospitalized patients in Spain in 2020. We also analyzed the risk factors for co-infection, the etiology and the impact in the outcome.

**Methods:**

Data were obtained from records in the Minimum Basic Data Set (MBDS) of the National Surveillance System for Hospital Data in Spain, provided by the Ministry of Health and annually published with 2 years lag. COVID-19 circulated in two waves in 2020: from its introduction to 31st June and from 1st July to 31st December. The risk of developing a healthcare-associated bacterial co-infection and the risk for in-hospital and intensive care unit (ICU) mortality in co-infected patients was assessed using an adjusted logistic regression model.

**Results:**

The incidence of bacterial co-infection in COVID-19 hospitalized patients was 2.3%. The main risk factors associated with bacterial co-infection were organ failure, obesity and male sex. Co-infection was associated with worse outcomes including higher in-hospital, in-ICU mortality and higher length of stay. Gram-negative bacteria caused most infections. Causative agents were similar between waves, although higher co-infections with *Pseudomonas* spp. were detected in the first wave and with *Haemophilus influenzae* and *Streptococcus pneumoniae* in the second.

**Conclusions:**

Co-infections are not as common as those found in other viral respiratory infections; therefore, antibiotics should be used carefully. Screening for actual co-infection to prescribe antibiotic therapy when required should be performed.

## Introduction

SARS-COV2 coronavirus infection responsible for coronavirus disease 2019 (COVID-19), is a major public health problem. Since its emergence in December 2019, it has caused more than 500 million cases and more than 6 million deaths worldwide.^1^ In Spain, one of the most affected countries in the European Union, it has caused more than 13 million cases and a total of 110 394 deaths.^1^

At the beginning of the pandemic, little was known about COVID-19 disease, so treatments and decisions were based on experience from previous pandemics caused by other respiratory viruses, such as influenza A(H1N1)pdm09 in 2009. That pandemic was characterized by high bacterial superinfection rates (30–55%)^2^ in patients infected with the virus that came with worse outcomes including; longer hospital stays, more intensive care unit (ICU) admissions and higher mortality.^3^ Therefore, antibiotic therapy was prescribed to more than 70% of COVID-19 patients for prevention.^4–6^ However, lower rates of co-infection (3–15%) have been reported in COVID-19 patients.^7^ In a study carried out by Garcia-Vidal^8^ in Spain including 989 patients, bacterial superinfection was only observed in 3% of cases. Another study conducted in England^9^ reported that only 6% of cases presented bacterial superinfection among 836 patients recruited. The results reported in COVID-19 contrast with those of other viral respiratory infections where co-infections and superinfections are often present.^3^^,^^10^

Although there are previous studies^8^^,^^11^^,^^12^ involving bacterial co-infections in COVID-19 patients, we propose a nationwide study that groups all COVID-19 hospitalized patients based on the National Surveillance System for Hospital Data.

The aim of the study was to describe the epidemiology of bacterial co-infections in COVID-19 hospitalized patients, their etiology and risk factors, and their impact in outcomes.

## Methods

### Study design and data source

A nationwide population-based retrospective study was performed in all COVID-19 hospitalized patients during the first year of the pandemic, 2020. Data were obtained from records in the Minimum Basic Data Set (MBDS) of the National Surveillance System for Hospital Data in Spain, provided by the Ministry of Health, annually published with 2 years lag. The MDBS is a clinical and administrative database, which has an estimated coverage of 99.5% of hospital discharges registered in both public and private hospitals in Spain.^13^^,^^14^ The MDBS includes 20 diagnoses each one with an indicator if the diagnosis was present on admission (POA), and 20 procedures according to the International Classification of Diseases 10th Revision, Clinical Modification (ICD-10-CM).^15^ The MDBS provides encrypted patient identification, sex, age, dates of hospital admission and discharge, intensive care unit (ICU) admission, length of ICU stay, diagnosis and procedures during hospitalization, as well as outcome at discharge. The MBDS is validated for data quality and methodology by the Spanish Ministry of Health, establishing protocols and periodic audits. The data were treated with full confidentiality according to Spanish legislation. Thus, given the anonymous and mandatory nature of the data, informed consent was not required or necessary. This study was approved by the Ethics Committee of Valladolid East Health Area under the code PI 22-2855.

### Study variables

COVID-19 was defined as the presence of ICD-10-CM codes B97.29 and U07.1 as main diagnosis and POA from 1st January to 31st December 2020.^16^ Sepsis was defined by the codes adapted from MacLaren et al.,^17^ Esper et al.,^18^ Dombrovskiy et al.^19^ and Bateman et al.^20^ (Supplementary table S1). In addition, severe sepsis was defined as the presence of a source of infection (Supplementary table S2) adapted from Esper et al.^18^and Wang et al.,^21^ and organ dysfunction (Supplementary table S3) according to the Angus sepsis abstraction criteria^22^ adapted by Shen et al.^23^  ^and Bateman et al.^^20^ All codes were updated to ICD-10-CM by our group.

Our COVID-19 hospitalized patients were divided in two groups based on the presence or absence of nosocomial bacterial co-infection, named BI group and NBI group, respectively. ICD-10-CM codes for causal agents are described in Supplementary table S4. Regarding this, we studied the impact of co-infections in hospitalized COVID-19 patients on in-hospital and ICU mortality. Also, in 2020 in Spain, two waves of COVID-19 were reported by the Ministry of health:^16^ the first wave since its introduction in Spain until 30th June 2020, and the second from 1st July 2020, until 31st December 2020. Based on that, we aimed to compare the co-infection profiles and outcomes of both of them.

### Statistical analysis

The results were reported as median (interquartile range) for continuous variables and as frequencies and percentages for categorical variables. Categorical data and proportions were analyzed using chi-squared test or Fisher’s exact test, as required. *t*-Test or Mann–Whitney U test was used to compare continuous variables. The risk of developing a health care-associated bacterial co-infection was analyzed by logistic regression model adjusted by the presence of organ failure and age, sex, tobacco, diabetes, obesity, endocrine and metabolic disorders, respiratory diseases, hypertension, heart disease, liver disease, renal disease and cancer. We also calculated the odds for in-hospital and ICU mortality in patients with COVID-19 diagnosis according to the presence of a health care-associated bacterial co-infection, by using logistic regression models adjusted by presence of bacterial co-infection, and age, sex, tobacco, diabetes, obesity, endocrine and metabolic disorders, respiratory diseases, hypertension, heart disease, liver disease, renal disease and cancer. Statistical analysis was performed using Python 3.9. All tests were two-tailed with *P*-values < 0.05 considered significant.

## Results

### Patient characteristics

**Figure 1:**
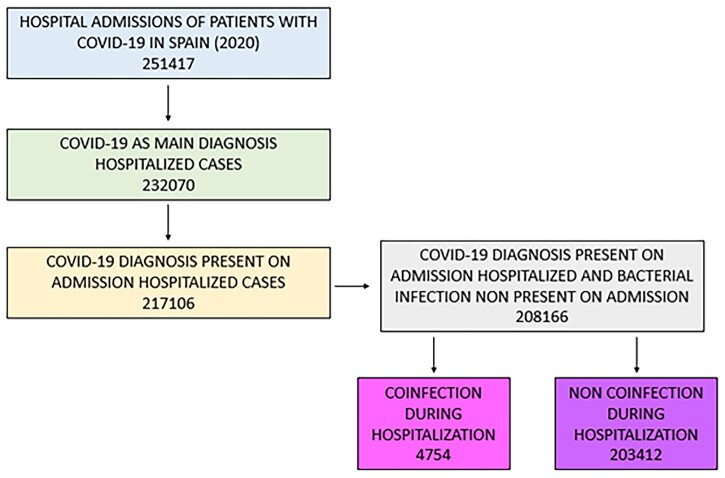
Study flowchart

We identified a total of 208 166 patients in Spain from 1st January to 31st December 2020 with a primary diagnosis of COVID-19 and present on admission. Of these, 4754 (2.3%) had an acute bacterial co-infection (figure 1). BI was present more often in males, patients had higher length of stay (LoS), in-hospital mortality, ICU admission, ICU mortality and ICU LoS, as well as a higher need for ventilatory support, compared to NBI patients. BI patients presented with higher obesity, heart disease and respiratory disease rates compared to NBI patients. In addition, more patients of the BI group developed sepsis or septic shock, and more than half presented an acute organ dysfunction, being respiratory, renal and hematologic the most frequent locations. The respiratory tract was the most frequent site of infection in both groups of study (table 1).

**Table 1:** Patient characteristics in COVID-19 hospitalized patients in Spain during 2020

	Total	NBI	BI	*P*-value
No.	208 166	203 412	4754	
Gender (male)	118 310 (56.83)	115 146 (56.61)	3164 (66.55)	<0.001
Mean age (years)	66.85 (66.77–66.92)	66.83 (66.76–66.91)	67.53 (67.16–67.89)	0.007
Length of stay (days)	10.62 (10.57–10.67)	9.93 (9.89–9.97)	40.04 (39.23–40.85)	<0.001
In-hospital death	34 206 (16.43)	32 616 (16.03)	1590 (33.45)	<0.001
Charlson Index	1.33 (1.32–1.34)	1.33 (1.32–1.34)	1.49 (1.44–1.54)	<0.001
No comoborbidities	100 788 (48.42)	98 787 (48.56)	2001 (42.09)	<0.001
1 comoborbidities	31 421 (15.09)	30 649 (15.07)	772 (16.24)	0.027
2 comoborbidities	35 789 (17.19)	34 874 (17.14)	915 (19.25)	<0.001
>2	40 168 (19.3)	39 102 (19.22)	1066 (22.42)	<0.001
ICU and intubation				
ICU	18 531 (8.9)	15 353 (7.55)	3178 (66.85)	<0.001
ICU death	5901 (31.84)	4718 (30.73)	1183 (37.22)	<0.001
ICU length of stay	15.44 (15.2–15.68)	12.3 (12.09–12.51)	30.6 (29.84–31.37)	<0.001
Mechanical ventilation	13 487 (6.48)	10 402 (5.11)	3085 (64.89)	<0.001
Ventilatory assistance	11 676 (5.61)	10 817 (5.32)	859 (18.07)	<0.001
Morbidities				
Abuse of tobacco	6803 (3.27)	6687 (3.29)	116 (2.44)	0.001
Diabetes	44 310 (21.29)	43 182 (21.23)	1128 (23.73)	<0.001
Obesity	20 017 (9.62)	19 404 (9.54)	613 (12.89)	<0.001
Endocrine and metabolic disorders	77 832 (37.39)	76 224 (37.47)	1608 (33.82)	<0.001
Respiratory diseases	31 088 (14.93)	30 297 (14.89)	791 (16.64)	0.001
Hypertension	86 312 (41.46)	84 401 (41.49)	1911 (40.2)	0.076
Heart disease	51 738 (24.85)	50 477 (24.82)	1261 (26.53)	0.007
Peripheral vascular disease	1273 (0.61)	1249 (0.61)	24 (0.5)	0.390
Liver disease	10 842 (5.21)	10 572 (5.2)	270 (5.68)	0.148
Renal disease	14 519 (6.97)	14 215 (6.99)	304 (6.39)	0.119
Cancer	9783 (4.7)	9563 (4.7)	220 (4.63)	0.840
HIV	451 (0.22)	442 (0.22)	9 (0.19)	0.801
Sepsis				
Sepsis	14 217 (6.83)	10 883 (5.35)	3334 (70.13)	<0.001
Sepsis + 1 organ failure	6776 (3.26)	4916 (2.42)	1860 (39.12)	<0.001
Sepsis + 2 organ failure	3305 (1.59)	2425 (1.19)	880 (18.51)	<0.001
Sepsis + >2 organ failure	1365 (0.66)	1047 (0.51)	318 (6.69)	<0.001
Organ failure				
Number of organ failure	0.7 (0.7–0.71)	0.69 (0.68–0.69)	1.35 (1.32–1.37)	<0.001
No organ failure	95 588 (45.92)	95 162 (46.78)	426 (8.96)	<0.001
1 organ	84 651 (40.67)	81 913 (40.27)	2738 (57.59)	<0.001
2 organ	22 956 (11.03)	21 767 (10.7)	1189 (25.01)	<0.001
>2 organ	4971 (2.39)	4570 (2.25)	401 (8.44)	<0.001
Organ failure				
Cardiovascular	2939 (1.41)	2652 (1.3)	287 (6.04)	<0.001
Hematologic	11 159 (5.36)	10 637 (5.23)	522 (10.98)	<0.001
Hepatic	7527 (3.62)	7191 (3.54)	336 (7.07)	<0.001
Neurologic	7857 (3.77)	7562 (3.72)	295 (6.21)	<0.001
Renal	22 844 (10.97)	22 351 (10.99)	493 (10.37)	0.186
Respiratory organ	90 199 (43.33)	86 105 (42.33)	4094 (86.12)	<0.001
Metabolic	3707 (1.78)	3333 (1.64)	374 (7.87)	<0.001
Site infection				
Nervous	34 (0.02)	32 (0.02)	2 (0.04)	0.406
Circulatory	250 (0.12)	226 (0.11)	24 (0.5)	<0.001
Respiratory	178 651 (85.82)	174 088 (85.58)	4563 (95.98)	<0.001
Digestive	963 (0.46)	663 (0.33)	300 (6.31)	<0.001
Genitourinary	8109 (3.9)	6042 (2.97)	2067 (43.48)	<0.001
Pregnancy	30 (0.01)	30 (0.01)	0 (0.0)	0.821
Skin soft tissue or bone	593 (0.28)	501 (0.25)	92 (1.94)	<0.001

### Bacterial infections and antimicrobial resistance

The comparison of BI patients in both waves is described in table 2. First, patients in the first wave had higher LoS in both general and ICU admission, as well as higher need of mechanical ventilation. However, patients in the second wave had higher in-hospital and ICU mortality, and higher need of ventilatory assistance. The profile of bacterial infections was similar in both waves, except for some agents. In the first wave, Gram-negative bacteria caused infections were higher, especially *Pseudomonas* spp. co-infections (42.08% vs. 38.02%). Meanwhile, in the second wave, *Streptococcus pneumoniae* (61.64%) and *Haemophilus influenzae* (3.80%) co-infections were present more often compared to the first wave (44.26 and 1.58%, respectively).

**Table 2:** Difference of microorganism specific rate linked to COVID-19 co-infection in both waves, incidence of antimicrobial resistance and the most common antimicrobial resistance in the BI group

	Total	1st wave	2nd wave	*P*-value
No.	4754	1576	3178	
Gender (male)	3164 (66.55)	1652 (67.37)	1512 (65.68)	0.228
Mean age (years)	67.53 (67.16–67.89)	66.94 (66.43–67.45)	68.16 (67.63–68.68)	0.001
Length of stay (days)	40.04 (39.23–40.85)	46.9 (45.59–48.21)	32.73 (31.91–33.55)	<0.001
In-hospital death	1590 (33.45)	714 (29.12)	876 (38.05)	<0.001
Charlson Index	1.49 (1.44–1.54)	1.41 (1.34–1.48)	1.58 (1.51–1.66)	0.001
No comoborbidities	2001 (42.09)	1105 (45.07)	896 (38.92)	<0.001
1 comoborbidities	772 (16.24)	399 (16.27)	373 (16.2)	0.980
2 comoborbidities	915 (19.25)	440 (17.94)	475 (20.63)	0.021
>2	1066 (22.42)	508 (20.72)	558 (24.24)	0.004
ICU and intubation				
ICU	3178 (66.85)	1655 (67.5)	1523 (66.16)	0.344
ICU death	1183 (24.88)	518 (21.13)	665 (28.89)	<0.001
ICU length of stay	30.6 (29.84–31.37)	34.99 (33.77–36.22)	25.84 (25.02–26.65)	<0.001
Mechanical ventilation	3085 (64.89)	1674 (68.27)	1411 (61.29)	<0.001
Ventilatory assistance	859 (18.07)	392 (15.99)	467 (20.29)	<0.001
Tipo bacteria familia				
Gram-positive bacteria	604 (12.71)	298 (12.15)	306 (13.29)	0.256
*Staphylococcus* spp.	479 (79.3)	240 (80.54)	239 (78.1)	0.461
*S. aureus*	115 (24.01)	49 (20.42)	66 (27.62)	0.065
Other Staphylococci	382 (79.75)	199 (82.92)	183 (76.57)	0.084
*Streptococcus* spp. and Enterococcus spp	134 (22.19)	61 (20.47)	73 (23.86)	0.317
*S. pneumoniae*	72 (53.73)	27 (44.26)	45 (61.64)	0.044
Other Streptococci	65 (48.51)	35 (57.38)	30 (41.1)	0.060
Other Gram-positive bacteria	1 (0.17)	1 (0.34)	0 (0.0)	0.310
Gram-negative bacteria	3862 (81.24)	2025 (82.59)	1837 (79.8)	0.015
*Haemophilus influenzae*	102 (2.64)	32 (1.58)	70 (3.81)	<0.001
*Neisseria meningitidis*	2 (0.05)	1 (0.05)	1 (0.05)	0.945
*Pseudomonas* spp.	1554 (40.24)	853 (42.12)	701 (38.16)	0.012
Enterobacterales	180 (4.66)	92 (4.54)	88 (4.79)	0.716
Other Gram-negative bacteria	537 (13.9)	294 (14.52)	243 (13.23)	0.247
Anaerobic bacteria	270 (5.68)	158 (6.44)	112 (4.87)	0.022
Other anaerobes	25 (9.26)	14 (8.86)	11 (9.82)	0.788
*Clostridium* spp.	248 (91.85)	145 (91.77)	103 (91.96)	0.955
Other bacterial infections	327 (6.88)	138 (5.63)	189 (8.21)	0.001
Mycoplasma pneumonia	16 (4.89)	6 (4.35)	10 (5.29)	0.696
Chlamydia pneumonia	5 (1.53)	3 (2.17)	2 (1.06)	0.417
Non-classified bacterial infections	306 (93.58)	129 (93.48)	177 (93.65)	0.950
Antimicrobial drug resistance	749 (15.76)	404 (16.48)	345 (14.99)	0.171
Resistance to beta-lactam antibiotics	551 (73.56)	300 (74.26)	251 (72.75)	0.642
Meticilin-resistant *S. aureus*	190 (25.37)	113 (27.97)	77 (22.32)	0.076
Resistance to penicilins	83 (11.08)	44 (10.89)	39 (11.3)	0.857
Resistance to cephalosporins	45 (6.01)	25 (6.19)	20 (5.8)	0.822
Resistance to other beta-lactam antibiotics	377 (50.33)	195 (48.27)	182 (52.75)	0.221
Resistance to glucopeptides	2 (0.27)	2 (0.5)	0 (0.0)	0.191
Resistance to quinolones	31 (4.14)	22 (5.45)	9 (2.61)	0.052
Resistance to aminoglycoside macrolides sulfonamides or tetracyclines	26 (3.47)	14 (3.47)	12 (3.48)	0.992
Resistance to other antibiotics	241 (32.18)	134 (33.17)	107 (31.01)	0.529
Resistance to multiple antibiotics	150 (20.03)	74 (18.32)	76 (22.03)	0.206

No differences in antimicrobial resistance profiles were found in COVID-19 hospitalized patients between waves.

### Risk of bacterial co-infection

Supplementary table S5 shows the adjusted risk of bacterial co-infection. The risk of bacterial co-infection risk was not influenced by the seasonality (second vs. first wave, aOR = 0.97, *P*-values = 0.370).

### In-hospital and ICU mortality risks due to bacterial co-infection

Next, we evaluated the risks of mortality associated with bacterial co-infection. Overall, bacterial co-infection was an important risk factor for both, in-hospital and ICU mortality (aOR 3.32 and 1.27, respectively) (Supplementary table S6).

**Figure 2:**
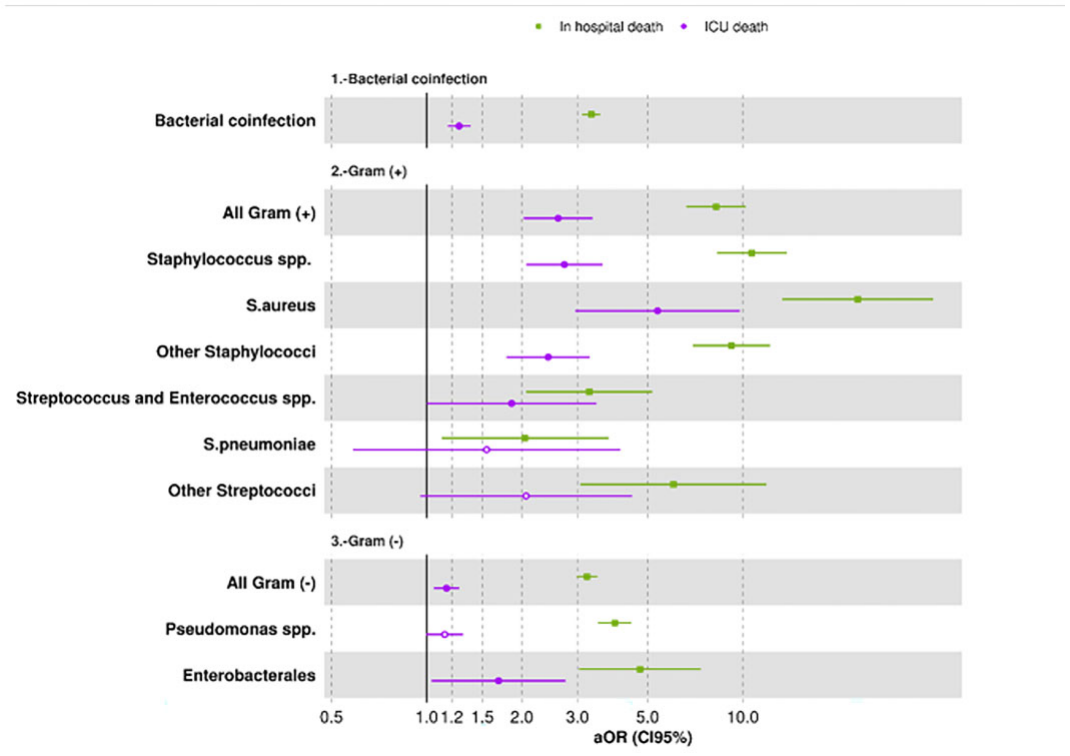
In-hospital and ICU mortality risk due to bacterial co-infections in COVID-19 patients

Risks according to bacterial classification was performed to evaluate which bacteria had the highest impact in mortality. Both Gram-positive and Gram-negative bacteria were associated with higher mortality, especially for in-hospital mortality in all cases (figure 2). Additionally, infections due to Gram-positive bacteria had a higher impact than infections caused by Gram-negative bacteria. Among Gram-positive bacteria, higher risks were found for Staphylococcal compared to Streptococcal infections, particularly for *Staphylococcus aureus* and in-hospital mortality (aOR 23.1). A co-infection with bacteria belonging to Enterobacterales outstands as a higher risk of in-hospital mortality (aOR 4.71) among Gram-negative bacteria.

## Discussion

In this retrospective study of COVID-19 hospitalized patients in Spain during 2020 with a nosocomial bacterial infection, we found that (i) the incidence of bacterial co-infection was 2,3% and was associated with worse outcomes; (ii) the main factors related with the development of a bacterial co-infection were the presence of an organ failure, obesity and male sex; (iii) Gram-negative bacteria were the most common causal agents of co-infection in both waves. *Pseudomonas* spp. was higher in the first wave compared to the second wave while *S. pneumoniae* and *H. influenzae* were higher in the second.

Many studies have tried to assess the incidence of bacterial co-infections in COVID-19 patients with different outcomes. A meta-analysis performed in 2020 concluded that 7% (95% CI 3–12) of the patients presented bacterial co-infection and raised up to 14% (95% CI 5–26) when it came to the ICU.^7^ However, another meta-analysis performed in 2021 estimated that the pooled bacterial co-infection prevalence reached up to 20.97%, showing great disparities between studies.^24^ In our study the prevalence of bacterial co-infection in hospitalized patients with COVID-19 is 2.3% and 17% in the case of ICU admitted patients in our country, which is lower than previously found for general admission, and higher for ICU admission. The biggest pandemic we have records of, occurred in 1918 caused by influenza A(H1N1) where mortality rates were so high due not only to the viral infection but to bacterial co-infections. Most recently, in 2009, another influenza pandemic caused by an emerging influenza A(H1N1) virus of swine origin (A(H1N1)pdm09) occurred and actually replaced the previously A(H1N1) circulating strain. This time mortality was not as high as 1918s thanks to the use of antibiotics and mechanical ventilation among others.^25^^,^^26^ The incidence of bacterial co-infections after viral pneumonia was 12% in hospitalized patients^27^ and 30% in patients admitted to the ICU.^28^ In the case of co-infections in COVID-19, the mechanisms that lead to such a lower incidence is not well understood. Several hypotheses have been made, including prophylactic antibiotic therapy at hospital admission and the presence of an immunological factor such as macrophage hyperactivation.^8^

The most frequent sites of infection were respiratory followed by urinary and digestive, probably due to catheter colonization and antibiotic use. The most often microorganisms found in co-infections were Gram-negative bacteria with higher prevalence of those, particularly *Pseudomonas* spp. in the first wave, and *S. pneumoniae* and *H. influenzae* in the second. The prevalence of one group over the other is a matter of controversy as Gram negatives predominate in some studies^29^^,^^30^ and Gram-positive bacteria in others.^31^ A study performed in a ICU unit in Spain also showed *Pseudomonas* spp. and *H. influenzae* to be among the most often found bacterias causing infection in COVID-19 patients.^32^ On the other hand, higher mortality risk was associated any bacterial co-infection with a higher risk in case of Gram-positive bacteria, which is probably due to the highest risk association of *S. aureus* co-infection. This microorganism is known for its virulence and has been previously associated with bacterial co-infection in patients with COVID-19 pneumonia along with *S. pneumoniae*, *H. influenzae* and *K. pneumonia.*^33^ Among Gram-negative bacteria, the enterobacterales presented the higher mortality risks which includes *K. pneumoniae* among others, which aligns with the aforementioned review.^33^

Gram-negative co-infections were higher in the first wave, especially *Pseudomonas* spp co-infections. One of the risk factors related to *Pseudomonas* spp. co-infection include mechanical ventilation which could lead to ventilator associated pneumonia.^34^ In the first wave, the patients required more mechanical ventilation^5^^,^^35^ which would explain the higher incidence of this pathogen. Instead, in the second wave, *S. pneumoniae* and *H. influenzae* co-infections were present more often compared to the first wave. *S. pneumoniae* and *H. influenzae* usually present seasonality with higher peaks in winter and spring,^36^^,^^37^ which will not explain why higher incidence was found in the second wave. However, it has been shown that circulation patterns of other pathogens have been modified due to the COVID-10 pandemic which could explain our results.^38^^,^^39^

Multiple factors have been associated with the risk of bacterial co-infections,^29^^,^^40^ such as advanced age, male sex and obesity which are consistent with our study. Organ failure is another factor we found significant. Regarding this, invasive techniques would be required in those cases which align with findings that associate higher risk with the use of catheters and invasive mechanical ventilation.^41^

Previous studies have shown bacterial co-infections upon viral pneumonia present with poorer outcomes.^42^^,^^43^ Additionally, studies have associated lymphocytopenia with COVID-19 severity and ICU admission. This condition is known to encourage the appearance of co-infections more frequently.^44^ Also, ICU-admitted patients require invasive measures and techniques that increase the susceptibility of nosocomial infections.^45^ Our results are consistent with previous studies and patients with BI had higher LoS, ICU admission and mortality than NBI patients. The higher LoS need for mechanical ventilation observed in the first wave could be reasonably explained by the lack of knowledge, the severity of the cases and the overwhelmed health system at the first stages of the pandemic. Whereas, in the second wave many admissions were made to follow-up in case of acute respiratory distress syndrome considerably reducing the LoS.^46^

Our study has limitations since we carried out a retrospective study using data obtained in the Spanish MBDS, with under coding of variables, lack of coding of analytical variables and lack of coding of several admissions of the same patient. The main advantage is the large size of the sample, which gives it high statistical power and allows us to provide a global view of the epidemiological situation of the entire Spanish population.

In conclusion, in this nationwide study, we report bacterial co-infections in 2.3% of COVID-19 hospitalized patients who presented higher in-hospital and ICU LoS and mortality, as well as ICU admission. The main microorganisms causing bacterial infections were Gram-negative bacteria, especially *Pseudomonas* spp*.* However, the higher mortality risks were found for Gram-positive bacteria, outstanding *S. aureus.*

Bacterial co-infections in COVID-19 hospitalized patients are not as common as found in other viral respiratory infections. Due to the low prevalence of bacterial co-infections, antibiotic therapy should be prescribed carefully to avoid complications derived from extensive use of antibiotics in these patients.

## Supplementary Material

ckad060_Supplementary_Data

## Data Availability

All data are available as a part of the article. Key points Coronavirus disease 2019 (COVID-19) infection is a public health problem that has caused more than 6 million cases worldwide. Bacterial co-infection in patients with COVID-19 increased the length of stay, the admissions to intensive care units and the mortality. Antibiotherapy should not be prescribed to all patients with COVID-19 infection. It is essential to detect a true co-infection in which case antibiotic therapy should be initiated.
