# Supplementary material for: Epidemiology of bacterial co-infections and risk factors in COVID-19-hospitalized patients in Spain: a nationwide study
Source: Eur J Public Health. 2023 Apr 22;33(4):675–81. doi: 10.1093/eurpub/ckad060 (PMC10393489; doi:10.1093/eurpub/ckad060)
Supplement: ckad060_Supplementary_Data [file ckad060_supplementary_data.docx]

Supplementary Material.

**References**

41. Cheng K, He M, Shu Q, Wu M, Chen C, Xue Y. Analysis of the Risk Factors for Nosocomial Bacterial Infection in Patients with COVID-19 in a Tertiary Hospital. Risk Manag Healthc Policy [Internet]. 2020 [cited 2022 Sep 14];13:2593–9. Available from: https://pubmed.ncbi.nlm.nih.gov/33223859/

42. Voiriot G, Visseaux B, Cohen J, Nguyen LBL, Neuville M, Morbieu C, et al. Viral-bacterial coinfection affects the presentation and alters the prognosis of severe community-acquired pneumonia. Crit Care [Internet]. 2016 Oct 25 [cited 2022 Sep 14];20(1). Available from: https://pubmed.ncbi.nlm.nih.gov/27852281/

43. Beadling C, Slifka MK. How do viral infections predispose patients to bacterial infections? Curr Opin Infect Dis [Internet]. 2004 Jun [cited 2022 Sep 14];17(3):185–91. Available from: https://pubmed.ncbi.nlm.nih.gov/15166819/

44. Diao B, Wang C, Tan Y, Chen X, Liu Y, Ning L, et al. Reduction and Functional Exhaustion of T Cells in Patients With Coronavirus Disease 2019 (COVID-19). Front Immunol [Internet]. 2020 May 1 [cited 2022 Sep 14];11. Available from: https://pubmed.ncbi.nlm.nih.gov/32425950/

45. Kollef MH, Torres A, Shorr AF, Martin-Loeches I, Micek ST. Nosocomial Infection. Crit Care Med [Internet]. 2021 Feb 1 [cited 2022 Nov 16];49(2):169–87. Available from: https://pubmed.ncbi.nlm.nih.gov/33438970/

46. Rees EM, Nightingale ES, Jafari Y, Waterlow NR, Clifford S, Carl CA, et al. COVID-19 length of hospital stay: A systematic review and data synthesis. BMC Med [Internet]. 2020 Sep 3 [cited 2022 Nov 16];18(1):1–22. Available from: https://bmcmedicine.biomedcentral.com/articles/10.1186/s12916-020-01726-3

| A02.1 | Salmonella septicemia |
| --- | --- |
| A20.7 | Septicemic plague |
| A22.7 | Septicemia due to anthrax |
| A39.4 | Meningococcal septicemia |
| A39.1 | Waterhouse-Friderichsen syndrome |
| A41.2 | Staphylococcal, unspecified |
| A41.0 | Sepsis due to Staphylococcus aureus |
| A41.1 | Sepsis due to other specified staphylococci |
| A40 | streptococcal sepsis |
| A40.3 | Sepsis due to Streptococcus pneumoniae |
| A41.4 | Sepsis due to anaerobes |
| A41.50 | Sepsis due to other gram-negative organisms |
| A41.3 | Sepsis due to Haemophilus influenzae |
| A41.51 | Escherichia coli sepsis [E. coli] |
| A41.52 | Pseudomonas sepsis |
| A41.53 | Sepsis due to Serratia |
| A41.59 | Sepsis due to other gram negatives |
| A41.89 | Other specified sepsis |
| A41.9 | Sepsis, unspecified organism |
| A54.86, A54.89 | Gonococcal septicemia |
| A48.3 | Toxic shock syndrome |
| B37.7 | Candida sepsis |
| B37.6 | Candidal endocarditis |
| B38.7 | Disseminated coccidioidomycosis |
| N39.0, B37.4, N30. N34, R82.81 | Urinary tract infection |
| R65 | Symptoms and signs specifically associated with systemic inflammation and infection |
| R78.81 | Bacteremia |
| T80.89 | Complication of medical care, other transfusion reaction |
| T81.1 | Postprocedural shock |
| T81.4 | Infection following a procedure |
| R65.* | Symptoms and signs specifically associated with systemic inflammation and infection |
| R57.* | Shock, not elsewhere classified |

**Supplementary table 1:** International Classification of Diseases, 10th Revision, Clinical Modification (ICD-10-CM) codes were used to identify sepsis caused by bacterial infections.

|  | **Nervous** |
| --- | --- |
| A17 | Tuberculosis of meninges and central nervous system |
| A39.0 | Meningococcal infection |
| A51.41 | Acute syphilitic meningitis (secondary) |
| A52.1 | symptomatic neurosyphilis |
| A52.2 | Asymptomatic neurosyphilis |
| A52.3 | Unspecified neurosyphilis |
| G00 | Bacterial meningitis, not elsewhere classified |
| G04.2 | Bacterial meningoencephalitis and meningomyelitis, not elsewhere classified |
| B45.1 | Cerebral cryptococcosis |
| G02 | Meningitis in other infectious and parasitic diseases classified elsewhere |
| G06 | Central nervous system abscess |
| G08 | Phlebitis of intracranial sinus |
| H44.0 | Purulent endophthalmitis |
| H05.00 | Acute inflammation of orbit |
| H60.20 | Malignant otitis externa |
| H70.0 | Acute mastoiditis |
|  | **Circulatory** |
| A52.00-A52.04 | Cardiovascular syphilis |
| A52.06 | Cardiovascular syphilis |
| A52.09 | Cardiovascular syphilis |
| A54.83 | gonococcal heart infection (endocarditis, pericarditis, myocarditis) |
| I01.2 | Acute rheumatic myocarditis |
| I30 | Acute pericarditis |
| I33 | Acute or subacute endocarditis |
|  | **Respiratory** |
| A15 | respiratory tuberculosis |
| A31.0 | Pulmonary diseases due to other mycobacteria |
| A36 | Diphtheria |
| A38 | Streptococcal throat/scarlet fever |
| A54.5 | Gonococcal infection of pharynx |
| B37.1 | Candidiasis, of lung |
| B38.0 | Acute pulmonary coccidioidomycosis |
| B38.1 | Chronic pulmonary coccidioidomycosis |
| B39.5 | Histoplasma duboisii pneumonia |
| B39.2 | Histoplasma capsulatum pneumonia |
| B39.9 | Histoplasmosis pneumonia unspecified |
| B45 | Cryptococcus neoformans |
| B44 | Aspergillosis |
| B59 | Pneumocystosis |
| J01 | Acute sinusitis |
| J02 | Acute pharyngitis |
| J03 | Acute tonsillitis |
| J04 | Acute laryngitis/tracheitis |
| J06 | Acute upper respiratory infection of multiple sites/not |
| J36 | Peritonsillar abscess |
| J12 | Viral pneumonia |
| J13 | Pneumococcal pneumonia |
| J15 | Other bacterial pneumonia |
| J16 | Pneumonia due to another specified organism |
| J18 | Pneumonia, organism not otherwise specified |
| J10 | Influenza |
| J44.1 | Acute exacerbation of obstructive chronic bronchitis |
| J47 | Bronchiectasis |
| J86 | Pyothorax |
| J85 | Abscess of lung and mediastinum |
|  | **Digestive** |
| A00 | Cholera |
| A01 | Typhoid/paratyphoid fever |
| A02 | Other salmonella infection |
| A03 | Shigellosis |
| A05 | Other food poisoning |
| A04.0-A04.4 | Intestinal infections due to *Escherichia coli* |
| A04.8 | Other specified bacterial intestinal infections |
| A04.9 | Bacterial intestinal infection, unspecified |
| A18.3 | Tuberculosis of intestines peritoneum and mesenteric glands |
| B82 | Intestinal parasitism unspecified |
| K04.7 | Periapical abscess without sinus |
| K04.6 | Periapical abscess with sinus |
| M27.2 | Inflammatory conditions of the jaw |
| K11.3 | Abscess of the salivary glands |
| K12.2 | Cellulitis and abscess of oral soft tissue |
| K35 | Acute appendicitis |
| K37 | Appendicitis not otherwise specified |
| K36 | Other appendicitis |
| K57.12 | Diverticulitis of the small intestine without haemorrhage |
| K57.13 | Diverticulitis of the small intestine with haemorrhage |
| K57.32 | Diverticulitis of colon without haemorrhage |
| K57.33 | Diverticulitis of colon with haemorrhage |
| K61.0 | Anal abscess |
| K61.1 | rectal abscess |
| K61.3 | Ischiorectal abscess |
| K65 | Peritonitis |
| K63.0 | Intestinal abscess |
| K94.02 | Infection of colostomy |
| K94.12 | Infection of enterostomy |
| K63.1 | Perforation of intestine |
| K75.0 | Abscess of liver |
| K75.1 | Portal pyaemia |
| K81.0 | Acute cholecystitis |
|  | **Genitourinary** |
| A18.1 | Tuberculosis of genitourinary system |
| A54.24 | Gonococcal salpingitis specified as acute |
| B37.4 | Candidiasis of other urogenital sites |
| N10 | Kidney infection |
| N39.0 | Urinary tract infection not otherwise specified |
| N41 | Prostatic inflammation |
| N45 | Orchitis and epididymitis |
| N73 | Female pelvic inflammation disease |
| N71 | Uterine inflammatory disease |
| N75.1 | Abscess of Bartholin’s gland |
| N76.4 | Other abscess of vulva |
|  | **Pregnancy** |
| O03 | Spontaneous abortion |
| O04 | Complications after (induced) termination of pregnancy |
| O08 | Complications following abortion and ectopic and molar pregnancies |
| O23 | Infections of genitourinary tract in pregnancy |
| O41.1 | Infection of amniotic cavity |
| O85 | Major puerperal infection |
| N61.1 | Abscess of breast |
|  | **Skin, soft tissue, or bone** |
| A02.24 | Salmonella osteomyelitis |
| A18.0 | Tuberculosis of bones and joints |
| A18 | Tuberculosis of other organs |
| A31.1 | Cutaneous diseases due to other mycobacteria |
| A146 | Erysipelas |
| A39.83 | Meningococcal arthropathy |
| A48.0 | Gas gangrene |
| A52.77 | Syphilis of bone |
| A54.5 | Gonococcal infection of joint |
| L03 | Cellulitis and acute lymphangitis |
| L05.01 | Pilonidal cyst, with abscess |
| L08 | Other local skin infection |
| M00 | Pyogenic arthritis |
| M72.6 | Necrotizing fasciitis |
| M86 | Osteomyelitis |
|  | **Other** |
| R78.81 | Bacteraemia |
| T79.8XXA | Post-traumatic wound infection, not elsewhere classified |
| T82.7 | Infection or inflammation of device/graft |
| T81. 4 | Postoperative infection |

**Supplementary table 2**: International Classification of Diseases, 10th Revision, Clinical Modification (ICD-10-CM) codes were used to identify the source of infection causing sepsis.

| **Organ System** | **ICD-10-CM Code** | **ICD-10-CM Code Description** |
| --- | --- | --- |
| **Cardiovascular** | I46 | Cardiac arrest |
|  | I95.1 | Orthostatic hypotension |
|  | I95.89 | Other specified hypotension |
|  | I95.9 | Hypotension, unspecified |
|  | R57.0 | Shock without mention of trauma |
|  | R57.9 | Hypotension, transient |
| **Hematologic** | D65 | Disseminated intravascular coagulation (Defibrination syndrome) |
|  | D68 | Other and unspecified coagulation defects |
|  | D69 | Purpura and other bleeding conditions |
|  | R79.1 | Abnormal coagulation profile |
| **Hepatic** | K72.0 | Acute and subacute necrosis of liver |
|  | K72.01, K72.91 | Hepatic encephalopathy |
|  | K75.9 | Hepatitis (septic & not elsewhere classified) |
|  | K76.3 | Hepatic infarction |
| **Neurologic** | F06 | Transient organic psychosis |
|  | G93.1 | Anoxic brain damage |
|  | G93.4 | Other and unspecified types of encephalopathy |
|  | G31.2 | Alcoholic encephalopathy |
|  | G94 | Other disorders of the brain in diseases classified elsewhere |
|  | I67.4 | Hypertensive encephalopathy |
|  | R40 | Drowsiness, stupor and coma |
|  | 4A00 | Electroencephalography |
| **Renal** | N00 | Acute glomerulonephritis |
|  | N17 | Acute renal failure |
|  | N19 | Renal shutdown, renal failure unspecified |
|  | 5A1D | Hemodialysis |
| **Respiratory** | J95 | Intraoperative and postprocedural complications and disorders of the respiratory system, not elsewhere classified |
|  | R06.81 | Apnea |
|  | R09.2 | Respiratory arrest |
|  | J96 | Respiratory insufficiency |
|  | 5A19, 5A09 | Ventilator management |
|  | [0BH1](javascript:loadCT(%220BH1%22,%22cie10pcs%22,%222012%22)) | Endotracheal intubation (emergency procedure) |
|  | A09357, 5A09557, 5A09457 | Continuous positive airway pressure |
| **Metabolic** | E87.2 | Acidosis, metabolic or lactic |

**Supplementary table 3**: International Classification of Diseases, 10th Revision, Clinical Modification (ICD-10-CM) codes for acute organ dysfunction

|  | GRAM-POSITIVE BACTERIA |  |
| --- | --- | --- |
| A41.0 | Sepsis due to *Staphylococcus aureus* | *S.aureus* |
| A41.1 | Sepsis due to other specified staphylococcus | Other Staphylococci |
| A41.2 | Sepsis due to unspecified staphylococcus | Other Staphylococci |
| A49.0 | Staphylococcal infection, unspecified site | Other Staphylococci |
| G00.3 | Staphylococcal meningitis | Other Staphylococci |
| M00.0 | Staphylococcal arthritis and polyarthritis | Other Staphylococci |
| J15.2 | Pneumonia due to *staphylococcus* | Other Staphylococci |
| A05.0 | Staphylococcal food poisoning | Other Staphylococci |
| J13.* | Pneumonia due to *Streptococcus pneumoniae* | *S.pneumoniae* |
| G00.1 | Pneumococcal meningitis | *S.pneumoniae* |
| M00.1 | Pneumococcal arthritis and polyarthritis | *S.pneumoniae* |
| G00.2 | Streptococcal meningitis | Other Streptococci |
| J15.3 | Pneumonia due to *streptococcus, group B* | Other Streptococci |
| J15.4 | Pneumonia due to other *streptococci* | Other Streptococci |
| A40.* | Streptococcal sepsis | Other Streptococci |
| A49.1 | Streptococcal and enterococcal infection, unspecified site | Other Streptococci |
| B95.* | *Streptococcus* and *staphylococcus* as the cause of diseases classified to other chapters | *Streptococcal* |
| A46.* | Erysipelas | Other Streptococci |
| A38.* | Scarlet fever | Other Streptococci |
| A42.* | Actinomycosis | *Other gram-positive bacteria* |
| A43.* | Nocardiosis | *Other gram-positive bacteria* |
|  | **GRAM-NEGATIVE BACTERIA** |  |
| A49.2 | *Haemophilus influenzae* infection, unspecified site | *Haemophilus influenzae* |
| A41.3 | Sepsis due to *Haemophilus influenzae* | *Haemophilus influenzae* |
| G00.0 | *Haemophilus meningitis* | *Haemophilus influenzae* |
| J14.* | Pneumonia due to *Haemophilus influenzae* | *Haemophilus influenzae* |
| B96.3 | Haemophilus influenzae [H. influenzae] as the cause of diseases classified elsewhere | *Haemophilus influenzae* |
| J15.1 | Pneumonia due to *Pseudomonas* | *Pseudomonas spp.* |
| B96.5 | *Pseudomonas (aeruginosa) (mallei) (pseudomallei)* as the cause of diseases classified elsewhere | *Pseudomonas spp.* |
| J15.0 | Pneumonia due to *Klebsiella pneumoniae* | *Enterobacterales* |
| A02.1 | Salmonella septicemia | *Enterobacterales* |
| A41.53 | *Serratia* | *Enterobacterales* |
| J15.5 | Pneumonia due to *Escherichia coli* | *Enterobacterales* |
| A01.* | Typhoid and paratyphoid fevers | *Enterobacterales* |
| A02.* | Other salmonella infections | *Enterobacterales* |
| A03.* | Shigellosis | *Enterobacterales* |
| B96.1 | *Klebsiella pneumoniae [K. pneumoniae]* as the cause of diseases classified elsewhere | *Enterobacterales* |
| B96.2 | *Escherichia coli [E. coli]* as the cause of diseases classified elsewhere | *Enterobacterales* |
| B96.4 | *Proteus (mirabilis) (morganii)* as the cause of diseases classified elsewhere | *Enterobacterales* |
| A04.0 | Infection due to enteropathogenic *Escherichia coli* | *Enterobacterales* |
| A04.1 | Infection due to enterotoxigenic *Escherichia coli* | *Enterobacterales* |
| A04.2 | Infection due to enteroinvasive *Escherichia coli* | *Enterobacterales* |
| A04.3 | Infection due to enterohemorrhagic *Escherichia coli* | *Enterobacterales* |
| A04.4 | Other intestinal infections due to *Escherichia coli* | *Enterobacterales* |
| A04.6 | Enteritis due to *Yersinia enterocolitica* | *Enterobacterales* |
| A04.5 | Enteritis due to *Campylobacter* | *Other gram-negative bacteria* |
| A00.* | Cholera | Other gram-negative bacteria |
| B96.82 | *Vibrio vulnificus* as the cause of diseases classified elsewhere | Other gram-negative bacteria |
| A05.3 | Food poisoning due to *Vibrio parahaemolyticus* | Other gram-negative bacteria |
| A05.5 | Food poisoning due to *Vibrio vulnificus* | Other gram-negative bacteria |
| B96.81 | *Helicobacter pylori [H. pylori]* as a cause of diseases classified elsewhere | Other gram-negative bacteria |
| A41.5 | Sepsis due to other Gram-negative organisms | Other gram-negative bacteria |
| J15.6 | Pneumonia due to other Gram-negative bacteria | Other gram-negative bacteria |
| A39.* | Meningococcal infection | *Neisseria meningitidis* |
| A44.* | Bartonellosis | Other gram-negative bacteria |
|  | **ANAEROBES** |  |
| A41.4 | Sepsis due to anaerobes | Other anaerobes |
| B96.6 | *Bacteroides fragilis [B. fragilis]* as a cause of diseases classified elsewhere | Other anaerobes |
| A05.4 | Food poisoning due to Bacillus cereus | Other anaerobes |
| B96.7 | *Clostridium perfringens [C. perfringens]* as a cause of diseases classified elsewhere | *Clostridium spp.* |
| A04.7 | Enterocolitis due to *Clostridium difficile* | *Clostridium spp.* |
| A05.1 | Food poisoning due to botulism | *Clostridium spp.* |
| A05.2 | Food poisoning due to *Clostridium perfringens [Clostridium welchii]* | *Clostridium spp.* |
|  | **OTHER BACTERIA INFECTIONS** |  |
| J15.7 | Pneumonia due to *Mycoplasma pneumoniae* | *Mycoplasma pneumoniae* |
| B96.0 | Mycoplasma pneumoniae [M. pneumoniae] as the cause of diseases classified elsewhere | *Mycoplasma pneumoniae* |
| J16.0 | *Chlamydial pneumonia* | *Chlamydia pneumoniae* |
|  | **OTHER BACTERIAL INFECTIONS** |  |
| G01.* | Meningitis in bacterial diseases classified elsewhere | Non-classified bacterial infections |
| G00.9 | Bacterial meningitis, unspecified | Bacterial infection |
| G00.8 | Other bacterial meningitis | Bacterial infection |
| B86.89 | Other bacterial agents as the cause of diseases classified elsewhere | Bacterial infection |
| A05.8 | Other food poisoning due to specified bacteria | Bacterial infection |
| A05.9 | Bacterial food poisoning, unspecified | Bacterial infection |
| J15.9 | Bacterial pneumonia, unspecified | Other gram-positive bacteria |

**Supplementary table 4:** ICD-10-CM codes for causal agents

|  | **aOR (CI95%)** | **p-value** |
| --- | --- | --- |
| **Age** |  |  |
| 0-19 | 0.12 (0.04-0.32) | <0.001 |
| 20-39 | 0.41 (0.34-0.51) | <0.001 |
| 40-59 | Reference |  |
| 60-79 | 2.01 (1.86-2.17) | <0.001 |
| ≥80 | 0.81 (0.73-0.9) | <0.001 |
| **Gender** |  |  |
| Female | Reference |  |
| Male | 1.46 (1.37-1.56) | <0.001 |
| **Comoborbidities** |  |  |
| No | Reference |  |
| Tobacco | 0.64 (0.53-0.77) | <0.001 |
| Obesity | 1.45 (1.33-1.58) | <0.001 |
| Cancer | 0.91 (0.79-1.04) | 0.172 |
| Liver disease | 1.02 (0.9-1.16) | 0.707 |
| Chronic respiratory disease | 1.16 (1.07-1.25) | <0.001 |
| Diabetes | 1.1 (1.02-1.18) | 0.011 |
| Endocrine and metabolic disorders | 0.76 (0.71-0.81) | <0.001 |
| Hypertension | 0.84 (0.79-0.9) | <0.001 |
| Renal disease | 0.94 (0.83-1.06) | 0.292 |
| Heart disease | 0.94 (0.88-1.01) | 0.077 |
| **Waves** |  |  |
| First wave | Reference |  |
| Second wave | 0.97 (0.92-1.03) | 0.370 |

**Supplementary table 5.** Adjusted odds ratios (aOR) and 95% confidence intervals for bacterial coinfection. aOR were computed by logistic regression.

|  | **OR(CI95%)** | |
| --- | --- | --- |
|  | **In-hospital death** | **ICU death** |
| **Bacterial co-infection** | 3.32 (3.10; 3.56) | 1.27 (1.16;1.38) |
| **All Gram (+)** | 8.25 (6.62;10.28) | 2.61 (2.03;3.35) |
| *Staphylococcus spp.* | 10.7 (8.25;13.74) | 2.72 (2.08;3.6) |
| *S.aureus* | 23.1 (13.33;40.04) | 5.37 (2.94;9.78) |
| Other Staphylococci | 9.21 (6.96;12.18) | 2.41 (1.79;3.29) |
| *Streptococcus* and *Enterococcus spp*. | 3.25 (2.08;5.16) | 1.86 (1.0;3.46) |
| *S.pneumoniae* | 2.05 (1.12;3.74) | 1.55 (0.58;4.1) |
| Other Streptococci | 6.05 (3.06;11.94) | 2.05 (0.95;4.44) |
| **All Gram (-)** | 3.22 (2.97;3.46) | 1.15 (1.05;1.27) |
| *Pseudomonas spp.* | 3.94 (3.49;4.44) | 1.14 (1.0;1.31) |
| Enterobacterales | 4.71 (3.03;7.39) | 1.68 (1.04;2.75) |

**Supplementary table 6.**  Risks of in-hospital and ICU mortality due to bacterial co-infection
